# Spiramycin-loaded maltodextrin nanoparticles as a promising treatment of toxoplasmosis on murine model

**DOI:** 10.1007/s00436-024-08280-4

**Published:** 2024-07-24

**Authors:** Ayman A. Abdel-Wahab, Dalia A. Shafey, Sahar M. Selim, Soraya A. Sharaf, Khloud K. Mohsen, Dina M. Allam, Sally W. Elkhadry, Marwa A. Gouda

**Affiliations:** 1 Department of Clinical and Molecular Parasitology, National Liver Institute, Menoufia University, Menoufia, Egypt; 2 Department of Pathology, Faculty of Medicine, Menoufia University, Shibin Elkom, Egypt; 3 Department of Epidemiology and Preventive Medicine, National Liver Institute, Menoufia University, Menoufia, Egypt

**Keywords:** Toxoplasmosis, Treatment, Spiramycin, Maltodextrin nanoparticles

## Abstract

Despite being the initial choice for treating toxoplasmosis, sulfadiazine and pyrimethamine have limited effectiveness in eliminating the infection and were linked to a variety of adverse effects. Therefore, the search for new effective therapeutic strategies against toxoplasmosis is still required. The current work is the first research to assess the efficacy of spiramycin-loaded maltodextrin nanoparticles (SPM-loaded MNPs) as a novel alternative drug therapy against toxoplasmosis in a murine model. Fifty laboratory-bred Swiss albino mice were divided into five groups: normal control group (GI, *n* = 10), positive control group (GII, *n* = 10), orally treated with spiramycin (SPM) alone (GIII, *n* = 10), intranasal treated with SPM-loaded MNPs (GIV, *n* = 10), and orally treated with SPM-loaded MNPs (GV, *n* = 10). Cysts of *Toxoplasma gondii *ME-49 strain were used to infect the mice. Tested drugs were administered 2 months after the infection. Drug efficacy was assessed by counting brain cysts, histopathological examination, and measures of serum CD19 by flow cytometer. The orally treated group with SPM-loaded MNPs (GV) showed a marked reduction of brain cyst count (88.7%), histopathological improvement changes, and an increasing mean level of CD19 (80.2%) with significant differences. SPM-loaded MNPs showed potent therapeutic effects against chronic toxoplasmosis. Further research should be conducted to assess it in the treatment of human toxoplasmosis, especially during pregnancy.

**Graphical Abstract:**

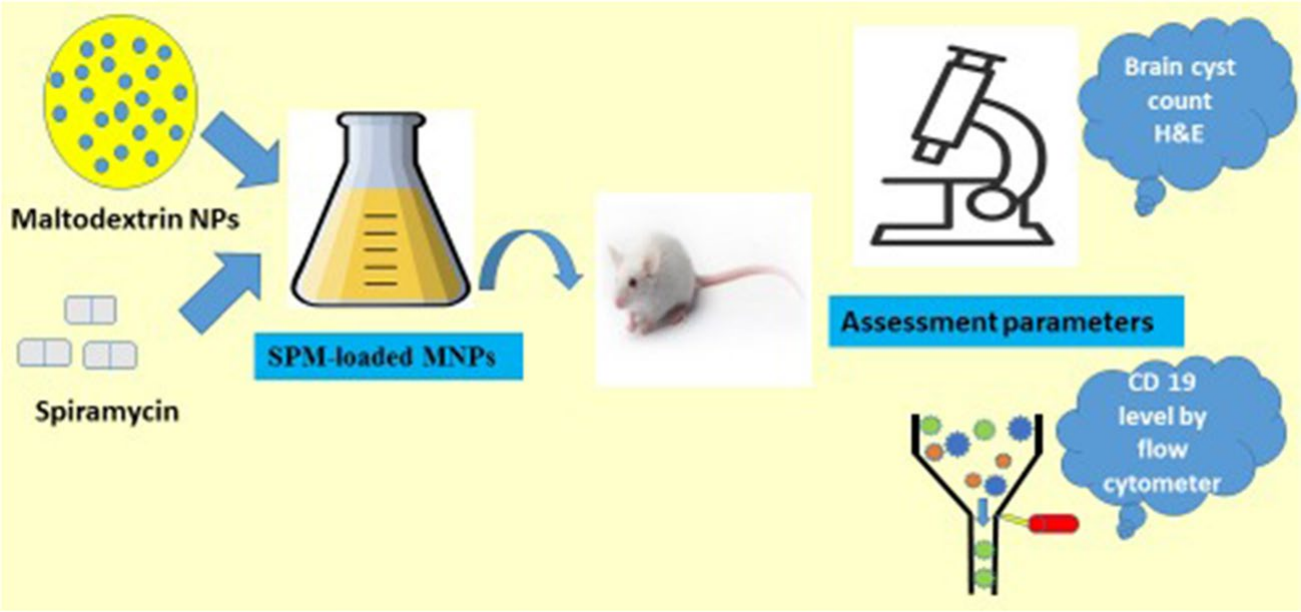

## Introduction

*Toxoplasma gondii* is a parasitic organism that has a complicated life cycle. It can infect all warm-blooded vertebrates, including humans. Domestic cats and other members of the Felidae family are the definitive hosts of this parasite, while humans and all other animals that are not felines are considered intermediate hosts (Hatam-Nahavandi et al. 2021).

Humans may acquire *T. gondii* infection by consuming food or water containing oocysts from cat feces or tissue cysts from undercooked infected meat (Dubey 2005). From the point of entry in the intestinal tract, *T. gondi* rapidly achieves systemic dissemination, and tachyzoites cross the blood–brain barriers (BBB) by using their gliding motility to actively invade neural cells where they replicate and provide an effective mechanism of propulsion inside tissues to finally establish latent infection in the central nervous system (CNS) and produce chronic cysts in the brain **(**Olivera et al. 2021).

The immune response against infection with toxoplasmosis plays a vital role in the resistance to the disease. The infection is controlled by inducing a potent immunity induced by TCD4 + and TCD8 + cells. In addition, the increased humoral immune response is mediated by CD19 lymphocyte proliferation, consequently increasing anti-*T. gondii* antibodies level that reduces the level of infection **(**Ribeiro et al. 2016).

*T. gondii* is one of the infrequent pathogens capable of traversing the placenta. The likelihood of transmitting the infection to the fetus through vertical transmission rises during pregnancy, with approximately 60 to 81% of *T. gondii* infections occurring in the third trimester. However, disease severity increases during the early stages of pregnancy depending on the parasite load and *T. gondii* genotype. (Manuel et al. 2020). Congenital toxoplasmosis can lead to fetal death, stillbirth, or long-term disabling sequelae (Moncada and Montoya 2012).

The clinical symptoms of toxoplasmosis differ based on the features of the parasite, including the strain’s virulence, in addition to host factors like genetic background and immunological status. A substantial fraction of the global population is infected, although it seldom leads to clinically severe illness. Nevertheless, specific individuals are prone to experiencing severe or life-threatening toxoplasmosis. The individuals in question encompass fetuses, neonates, and patients with compromised immune systems, for whom *T. gondii* can give rise to various clinical disorders (Mose et al. 2020).

Pyrimethamine is a dihydrofolate reductase inhibitor commonly administered as an effective treatment for toxoplasmosis. Additionally, trimethoprim and sulfamethoxazole were used as therapeutic agents for this illness. Most recommended medications were efficacious against the tachyzoite stage but not the cysts-harboring bradyzoites (Al-Malki 2021). Moreover, it is linked to many adverse consequences, including bone marrow toxicity, megaloblastic anemia, and pancytopenia (Saad et al. 2023). Chemotherapy for toxoplasmosis lacks specificity and efficacy against both stages of the parasite. It can only prevent the reproduction of the rapidly multiplying forms of the parasite, called tachyzoites. Still, it does not eliminate the dormant forms known as bradyzoites enclosed within protective tissue cysts. Hence, there is a need to explore novel therapeutic approaches to treat toxoplasmosis (El Sharazly et al. 2023).

Research has demonstrated that spiramycin exhibits promising efficacy against acute toxoplasmosis. Furthermore, it possesses lower levels of toxicity and attains larger concentrations in the placenta compared to alternative medications. This attribute aids in the prevention of parasite transmission from the mother to the fetus during pregnancy. Although spiramycin has remarkable benefits, it exhibits little ability to cross BBB. Hence, it is imperative to do additional research and advancements in the field of spiramycin to fully capitalize on its inherent benefits (Hagras et al. 2022).

Nowadays, nanotechnology is becoming a common approach in the treatment of toxoplasmosis. The synergy between nanotechnology and anti-*Toxoplasma* therapies presents an encouraging pathway towards safer, more efficient, and targeted treatments for combating toxoplasmosis (Ismael 2023).

Successful drug delivery is ensured by nanotechnology via improving the drug bioavailability and permeability through biological barriers, such as BBB, and lowering the drug doses required (Hagras et al. 2022). Maltodextrin is a starch hydrolysate composed of dextrose monomers connected by α-glycosidic linkages that have been growingly investigated for drug delivery and biomedical applications. They combine multiple merits such as high stability, safety, biocompatibility, and biodegradability. Moreover, they have sustainable and naturally abundant resources with low processing costs, rendering them environmentally and economically favorable materials (Abdel-Hafez et al. 2022).

## Materials and methods

### Animals and ethics statements

All animal studies were performed according to The ARRIVE guidelines, and after being approved by the ethical committee of the National Liver Institute (NLI IRB 00003413 FWA0000227). A total of 50 Swiss albino mice, reared in a laboratory setting, were acquired from the Theodor Bilharz Research Institute (TBRI). These mice had an average weight of roughly 20–25 g. The animals were housed in plastic cages with white wood chips as bedding in the animal facility of TBRI. They were provided unrestricted access to a commercial complete meal mixture and tap water. The lighting and temperature in their environment were strictly regulated, maintaining a temperature of 25 ± 2 °C (Abdallah et al. 2022).

### Experimental design

Mice were divided into five groups: normal control group (GI, *n* = 10), infected untreated positive control group (GII, *n* = 10), infected then orally treated with spiramycin alone group (GIII, *n* = 10), infected then intranasally treated with SPM-loaded MNPs group (GIV, *n* = 10), and infected then orally treated with SPM-loaded MNPs group (GV, *n* = 10).

### Tested drugs

Spiramycin (SPM): 3 M.I.U. (704 mg) spiramycin tablets [Rovamycin® -pharaonia Pharmaceuticals Company, Egypt] were crushed and then dissolved in distilled water to make oral suspension according to Rashed et al. (2022).

To prepare SPM-loaded maltodextrin nanoparticles (MNPs): First, maltodextrin was prepared according to Ducournau et al. (2017) then (0.5%, w/w) of the three polysaccharides; tea polysaccharide (TP), pumpkin polysaccharide (PP), and balsam pear polysaccharide (BP) were dissolved in the maltodextrin solution. The MNPs loaded with TP, PP, and BP were called MNPs-TP, MNPs-PP, and MNPs-BP. The emulsifier Tween 80 was chosen for the solution, and spiramycin was added to the previously prepared solution at a concentration of 100 mg/mL. One part of absolute ethanol was added to a mixed solution of MNPs/SPM with a volume ratio of 1:10. The SPM-loaded nanoparticles were acquired using centrifugation and subsequently washed with 100% ethanol on three occasions. The supernatants were combined and measured using spectrophotometry to determine their quantity. The concentration of the unknown SPM was measured by utilizing a standard curve generated from various concentrations of the SPM. The loading efficiency (LE) was determined using the following equation:

$$\text{LE}\%= \frac{A-B}{A}\times 100$$

A: Total content of SPM
B: Content of SPM in supernatant

SPM-loaded nanoparticles were administered at a dose of 100 mg/kg per day for 7 days according to Rashed et al. (2022).

### Infection of mice and treatment with the studied drugs

The mice were infected by intraperitoneal injection with 0.1mL brain suspension containing 1 × 10^2^ cysts per mL from previously infected mice with *Toxoplasma gondii *ME-49 strain (Etewa et al. 2018).

Treatment was administered 2 months after starting infection of the mice to all study groups except the negative control group was instead inoculated with phosphate buffer solution (PBS). SPM alone was administered orally (GIII) for 7 days via an esophageal tube at a single dose of 100 mg/kg/day per day for 7 days. SPM-loaded MNPs were administered at a single dose of 100 mg/kg/day per day for 7 days nasally (GIV) and orally (GV) (Rashed et al. 2022).

The mortality rate was recorded, and all surviving mice were euthanized with inhaled isoflurane and sacrified by cervical dislocation then dissected to obtain the brain, liver, and spleen. The brains were divided into two halves. The number of brain cysts was counted in one half, while the other half of each brain and other tissue samples were preserved in 10% formalin and stained with hematoxylin and eosin for histological investigation.

### Detection of *Toxoplasma* in the brain

Each brain hemisphere was carefully crushed in a mortar and pestle. Brain emulsion homogenates were acquired by combining crushed brain tissue with 1 mL of 10% formalin. A volume of 100 μL of brain homogenate was deposited onto a glass slide and inspected using a light microscope. The mean number of cysts in each group was calculated using the formula: cyst count in 100 μL × 10 × 2 (Abdallah et al. 2022).

### Histopathological assessment

The brain, liver, and spleen were preserved in 10% buffered formalin, dehydrated using different alcohol concentrations, and then treated with xylol for cleaning. Finally, they were individually embedded in paraffin blocks. The tissue sections of 5-µm thickness were dewaxed and stained with H&E. Subsequently, they were seen under a light microscope to identify any pathological alterations (Yahia et al. 2022). The sections were examined with a light microscope (with 10, 20, 40 and 100 × objectives), and pathological lesions in brain tissue were qualitatively classified for the presence of the following parameters: meningitis, perivascular cuffs, inflammatory cell infiltration, necrosis, hemorrhage, and gliosis according to Evangelista et al. (2021). The liver and spleen were examined for degree of inflammation, congestion, and necrosis. Pathological lesion severity was scored using the following scheme: 0, no lesion; 1, mild lesion; 2, moderate lesion; and 3, severe lesion. Pathological scores ranging from 0 to 3 were determined for the degree of inflammation and necrosis, according to Evangelista et al. (2023).

### Measurement of CD19 by flow cytometer

Mice blood samples were collected. Fluorophore-conjugated antibodies CD19 PE (Abenomics, USA) were added to 100 μL of whole unlysed blood. After sample processing according to Robinson (2022), cells were gated, and the data was analyzed using flow cytometry (Beckman Coulter Navios, Ireland). The histograms of logarithmic dot plots represent the lymphocyte population, and gating was performed on the CD19 positive cells. Parametric analysis as seen in flow cytometric images showed the percentage of CD19 population among the total lymphocyte cells.

### Statistical analysis

The data was encoded and inputted utilizing the Statistical Package for Social Science (SPSS) for Windows version 20. The data was depicted utilizing the measures of central tendency, specifically the mean, and the measure of dispersion, specifically the standard deviation. The Kruskal–Wallis test was employed to ascertain whether statistically significant disparities exist among the groups. A post hoc test (specifically, the Dunn-Bonferroni test) was utilized as a multiple comparison test to evaluate the differences between the groups. Values were deemed statistically significant if the *P* value was below 0.05.

The reduction rate of brain cyst count was calculated using the formula: (Mean value of brain cyst count in the untreated infected group minus the mean value of brain cyst count in the treated infected group) multiplied by 100, divided by the mean value of brain cyst count in the untreated infected group.

## Results

### Mortality rate and mice survival

**Fig. 1:**
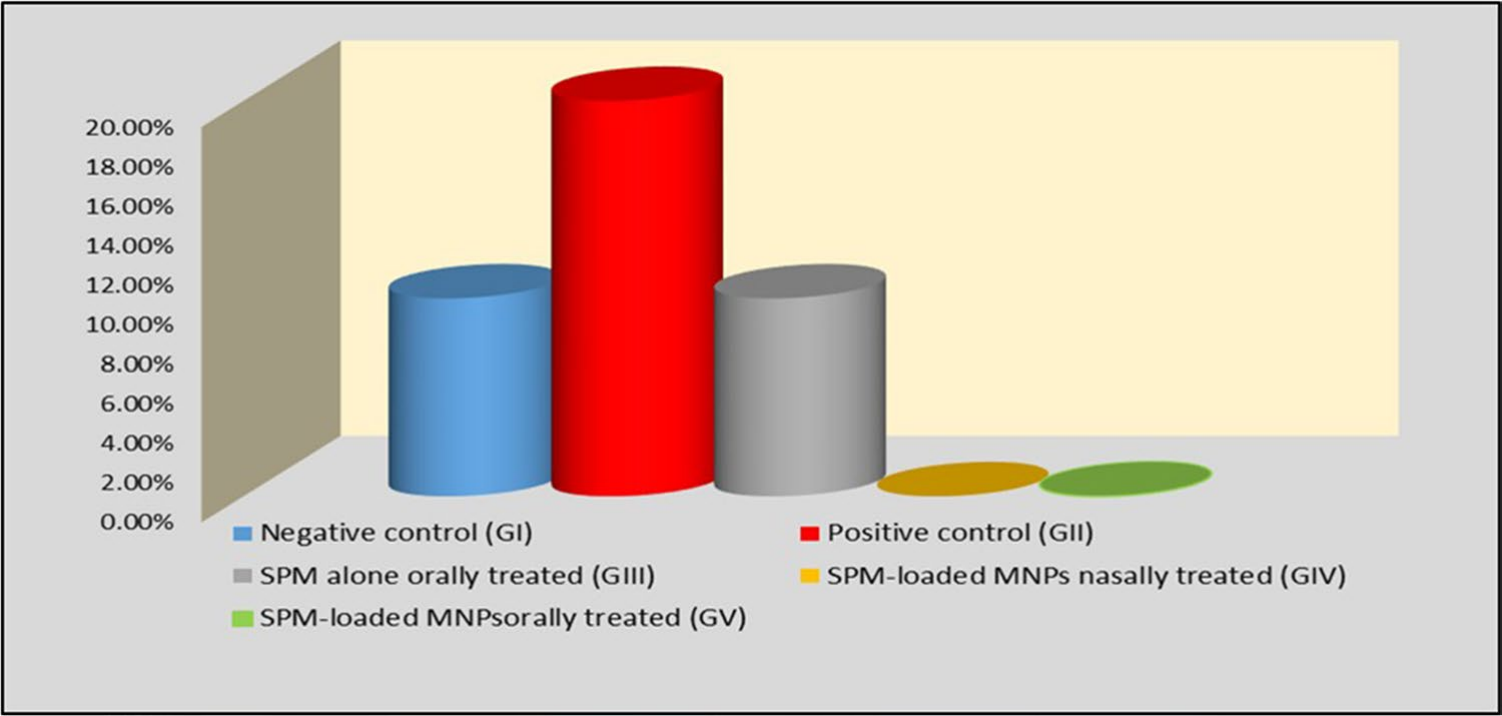
Mortality rate in the studied groups

The mortality rate was 20.0% in the positive control group, 10% in both the negative control and SPM orally treated alone group, while no mortality with 100% survival rate was detected among SPM-loaded MNPs nasally treated group and orally treated group (*P* > 0.05) (Fig. 1).

### The reduction rate of brain cyst count

The positive control group (GII) showed the highest mean number of brain cysts among the studied groups (257). Chronically infected mice orally treated with SPM-loaded MNPs (GV) accomplished the best results concerning brain cyst count reduction (88.7%) followed by SPM alone orally treated group (GIII) 70.8%, and the lowest reduction rate 65.4% was in nasally treated SPM-loaded MNPs (GIV) (*P*<00.05) (Table 1).

**Table 1:** Comparison of brain cyst counting mean level and reduction percentage among the studied groups

Count of cysts in the brain	Groups					Kruskal–Wallis test	*P* value
	Negative control (GI)	Positive control (GII)	SPM alone orally treated (GIII)	SPM-loaded MNPs nasally treated (GIV)	SPM-loaded MNPs orally treated (GV)		
Mean ± SD	0.0 ± 0.0	257.33 ± 44.7	75.33 ± 33.0	77.0 ± 87.78	29.33 ± 27.85	27	0.0001
Reduction rate			70.8%	65.4%	88.7%		

### Histopathological evaluation

#### Brain examination

**Fig. 2:**
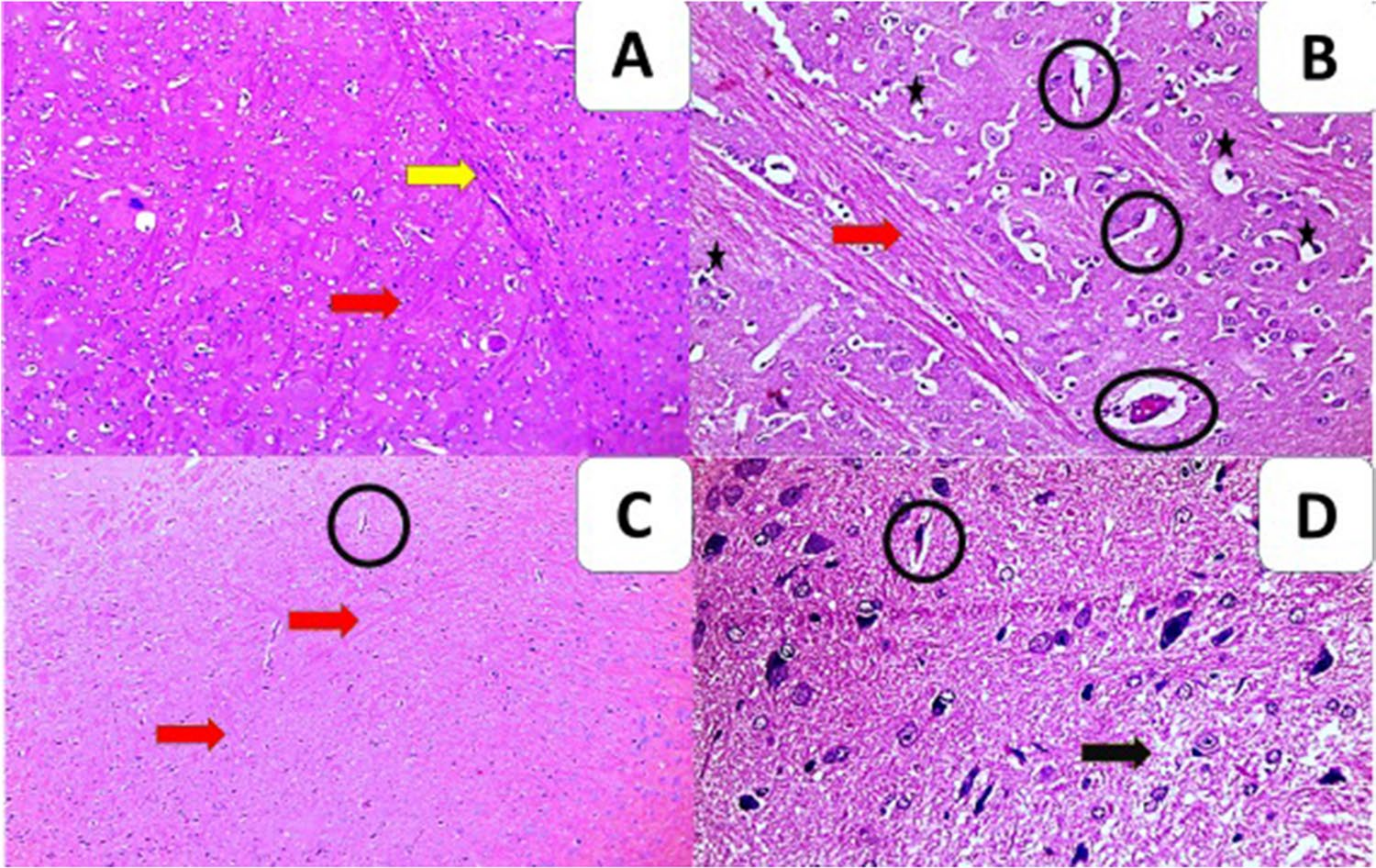
Cross sections of the brain (**A**, **B**, **C**, **D**). **A **Brain sections of the positive control group showed severe inflammatory reaction (yellow arrow) and severe neurodegenerative changes (red arrow) (H&E × 100). **B** SPM alone orally treated group (GIII) showed severe neurodegenerative changes (red arrow), severe vasculitis (circles), and apoptotic figures (stars) (H&E × 200). **C** SPM-loaded MNPs orally treated group (GV) showed mild inflammatory infiltration of mononuclear cells (lymphocytes), mild gliosis, mild neurodegenerative changes (red arrow), and mild vasculitis (circles) (H&E × 100). **D** SPM-loaded MNPs orally treated group (GV) high power showed edema (black arrow) and mild vasculitis (circles) (H&E × 200)

Positive control group exhibited extensive histopathological changes as severe meningeal inflammation and moderate vasculitis, presence of granuloma, moderate degree of gliosis, and presence of edema, whereas orally treated with SPM-loaded MNPs group showed the best improvement of the pathological changes with the absence of granuloma in the brain, mild neurodegenerative changes, mild degree of gliosis with only presence of edema (*P*<0.05) (Fig. 2).

#### Liver examination

**Fig. 3:**
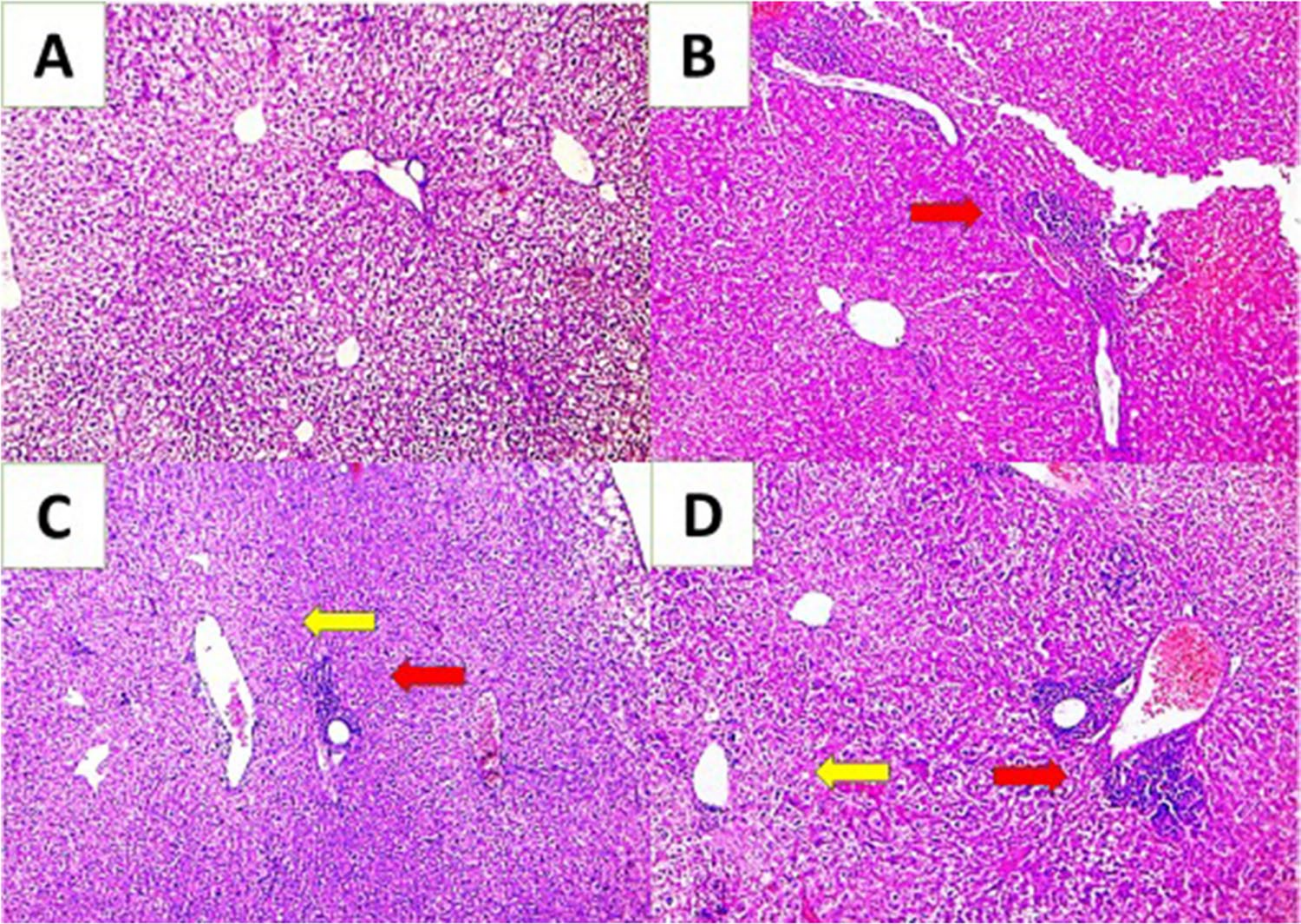
Cross sections of liver (**A**, **B**, **C**, **D**). **A** Normal liver section. **B** Liver sections of the positive control group showed vascular congestion, moderate portal inflammation and necrosis (red arrow). **C** SPM alone orally treated group (GIII) showed mild presence of portal inflammation (red arrow) and severe central vein necrosis and inflammation (yellow arrow). **D** SPM-loaded MNPs orally treated group (GV) showed moderate presence of portal inflammation (red arrow) and severe central vein necrosis and inflammation (yellow arrow) (H&E × 200).

Histopathological examination of hepatic tissues of the positive control group showed moderate presence of portal inflammation and central vein necrosis and inflammation. Among the treated groups, SPM alone orally treated group (GIII) showed mild presence of portal inflammation and severe central vein necrosis and inflammation, but SPM-loaded MNPs nasally and orally treated group showed moderate presence of portal inflammation and severe central vein necrosis and inflammation (*P*<0.05) (Fig. 3).

#### Spleen examination

**Fig. 4:**
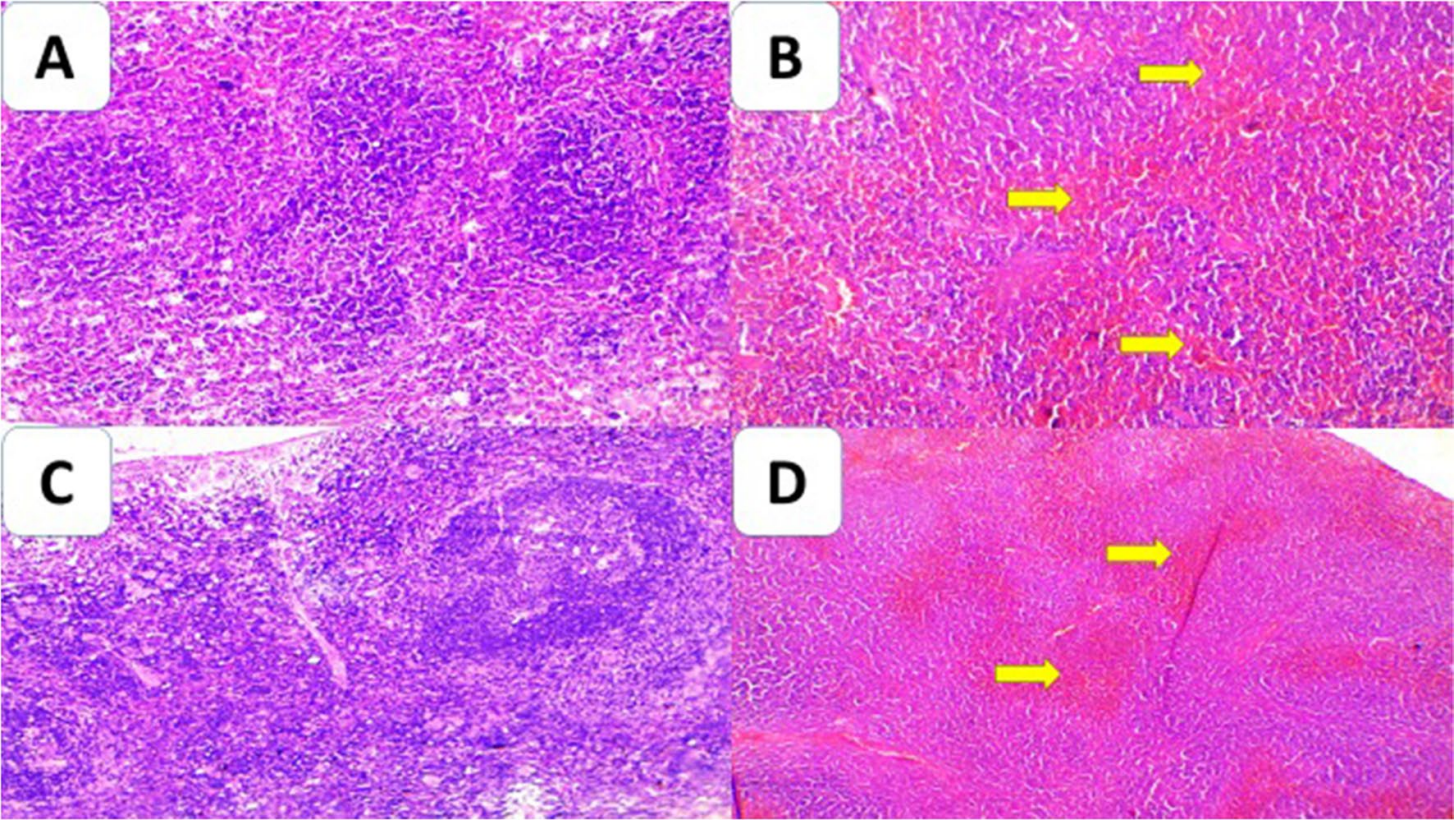
Cross sections of spleen (**A**, **B**, **C**, **D**). **A** Section of normal splenic follicles. **B** The positive control group showed atrophy of white pulp, fusion of follicles, and congestion of red pulp (red arrow). **C** SPM-loaded MNPs nasally treated group showed mild atrophy of white pulp and mild congestion of red pulp. **D** SPM-loaded MNPs orally treated group showed mild atrophy of white pulp and severe congestion of red pulp (yellow arrow) (H&E ×200).

All mice (100%) of the positive control group showed fusion of follicles, the presence of necrotic foci, and severe congestion. Among the treated groups, SPM alone orally treated group showed fusion of follicles, absence of necrotic foci, and mild presence of inflammatory cells in 90% of mice. The best improvement of the pathological changes was in the loaded MNPs-SPM nasally treated group that showed mild atrophy of white pulp, mild congestion, and mild inflammation. SPM-loaded MNPs orally treated group showed mild atrophy of white pulp, severe congestion and mild inflammation (*P*<0.05) (Fig. 4).

### Results of assessment of CD19 by flow cytometer

**Fig. 5:**
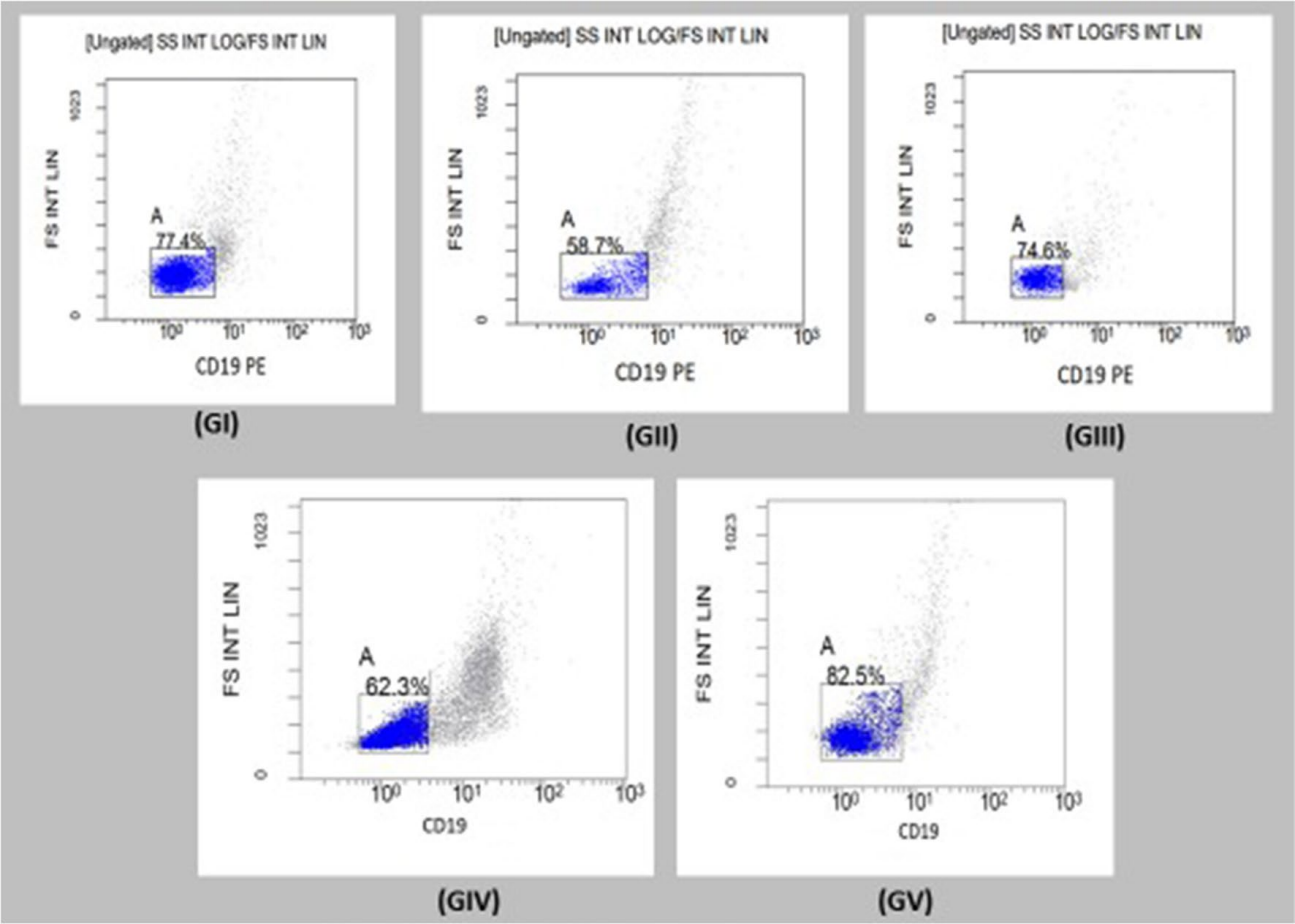
Histograms of logarithmic dot plots present the lymphocyte population and gating that was performed on the CD19-positive cells of samples of the different studied groups

The mean level of CD19 among the negative control group (GI) was 79.93, whereas mean level of CD19 was 68.3 in positive control group (GII), 72.87 in SPM alone orally treated group (GIII), and 66.58 in nasally treated SPM-loaded MNPs group (GIV). Increased mean level of CD19 in orally treated with SPM-loaded MNPs group (GV) that was 80.02 (*P*<0.05) (Table 2) (Fig. 5).

**Table 2:** Assessment of CD19 levels by flow cytometer in the studied groups

CD19 flow cytometer	Groups					Kruska-Wallis test	*P*-value
	Negative control (GI)	Positive control (GII)	SPM orally treated (GIII)	SPM-loaded MNPs nasally treated (GIV)	SPM-loaded MNPs orally treated (GV)		
Mean ± SD	79.93 ± 2.7	58.3 ± 4.7	72.8 ± 3.5	66.58 ± 7.09	80.02% ± 2.2*	24.3	0.0001

*Statistically significant difference (*P* = 0.003) between SPM-loaded MNPs orally treated group and positive control group using pair comparison test

## Discussion

Until now, chemotherapy for toxoplasmosis is not effective against all stages of the parasite. It can only inhibit the replication of the tachyzoites but cannot eradicate the bradyzoites which are surrounded by tissue cysts. Therefore, the search for new effective therapeutic strategies for toxoplasmosis is still required (El Sharazly et al. 2023). Several studies have investigated maltodextrin nanoparticles as nanocarriers in vaccination against toxoplasmosis (Ducournau et al. 2020; Fasquelle et al. 2023). Therefore, our study is the first to assess the efficacy of maltodextrin nanoparticles as drug delivery of spiramycin for the treatment of toxoplasmosis on the murine model.

Regarding the mortality rate that was recorded in our study, SPM-loaded MNPs nasally and orally treated groups exhibited the highest survival with no mortality rates until the end of the study. Our findings were consistent with those reported by Cheraghipour et al. (2020) who treated a group of mice with SPM-loaded chitosan nanoparticles (SLCNs). They had the highest survival up to 18 days after treatment and there was no mortality until the 8th day.

In the present work, a significant reduction of brain cyst count was higher (88.7%) in the orally treated group with SPM-loaded MNPs as compared to SPM alone orally treated group (70.8%), and the lowest reduction rate of 65.4% was in nasally treated SPM-loaded MNPs, which agreed with Etewa et al. (2018) study who recommended the use of SLCNs in the treatment of toxoplasmosis, evidenced by the high achieved reduction rates in parasite count induced in infected mice.

Hagras et al. (2019) also tested SLCNs in comparison with spiramycin alone for treatment of *T. gondii* revealed that SLCNs showed the highest significant reduction of tachyzoites in the brain, spleen, and liver denoting increasing efficacy of spiramycin and successful bypass of BBB. Correspondingly, the results were parallel with Keyhani et al. (2020) who treated mice with selenium nanoparticles (SeNPs); the mean number of brain tissue cysts decreased compared to the control group. As a result, SeNPs have preventive effects against latent toxoplasmosis with no significant toxicity.

For a more comprehensive evaluation of the efficacy of SPM-loaded MNPs, tissue sections from the brain, liver, and spleen were examined. The histopathological sections of the examined tissues showed great improvement after oral treatment of infected mice with spiramycin-loaded MNPs in comparison to the infected untreated control or infected mice group treated with spiramycin alone.

Our findings are consistent with Allam et al. (2022) who noticed significant tissue regenerative effects in the brain, liver, and spleen with the absence of tachyzoites in mice treated with SLCNs. It was also agreed with Sarhan et al. (2024) who revealed that zinc oxide and magnesium-doped zinc oxide nanoparticles significantly decreased the number of brain cysts by 29.30% and 35.08%, respectively, compared to the infected untreated group with marked histopathological improvement in the brain, liver, and spleen. They finally concluded that these nanoparticles have antiparasitic and antiapoptotic effects and could be used as adjuvants in treating chronic toxoplasmosis.

The mean level of CD19 was also evaluated in our research as an indicator for increasing immune response; infected untreated mice showed a decreased mean level of CD19 (68.3) and CD19 level was 72 in SPM alone orally treated group. On the other hand, infected mice orally treated with the SPM-loaded MNPs group showed an increased mean level of CD19 (80.02). This was following Wakid et al. (2023) who found that palm date extracts loaded with selenium nanoparticles induced immunomodulatory and anti-inflammatory effects against *T. gondii*. Hamad et al. (2020) also evaluated the synergistic effects of some nanoparticles and spiramycin on immune responses against toxoplasmosis and detected those mice receiving a combination of chitosan nanoparticles and silver nanoparticles loaded with spiramycin showed more improvements than single therapy.

Our study agreed with Hamad et al. (2018), who treated RH strain-infected mice with SLCNs on mice treated for 7 days and detected an increase in cytokines such as INF-γ and TNF-α and an increase in IgM levels that denoted improvement and enhancement of the immune response_._ This contrasted with Ismael’s (2023) study which concluded that nanoparticles are hazardous to the parasite but not to the host cells both in vitro and in vivo; however, they can interfere with pregnancy and may limit their usefulness.

## Conclusion marks

Spiramycin when loaded on maltodextrin nanoparticles and administered orally leads to a marked reduction of brain cyst count and increases the efficacy of spiramycin in the treatment of toxoplasmosis. Thus, it could be a promising treatment for toxoplasmosis due to its capability to penetrate the tissues, particularly the BBB. However, extensive in vivo and clinical trials are needed to approve the effects of SPM-loaded MNPs as a treatment of human toxoplasmosis especially during pregnancy.

## Data Availability

No datasets were generated or analysed during the current study.

## Abbreviations

SPM-loaded MNPs: Spiramycin-loaded maltodextrin nanoparticles
SPM: Spiramycin
BBB: Blood-brain barriers
CNS: Central nervous system
TBRI: Theodor bilharz research institute
IRB: Institutional review board
TP: Tea polysaccharide
PP: Pumpkin polysaccharide
BP: Balsam pear polysaccharide
SPSS: Statistical package for social science
SLCNs: SPM-loaded chitosan nanoparticles
SeNPs: Selenium nanoparticles
